# Safety competence promotion in secondary education – A case of the Finnish NouHätä! Programme

**DOI:** 10.1016/j.heliyon.2024.e28099

**Published:** 2024-04-12

**Authors:** Mikko Puolitaival, Brita Somerkoski, Eila Lindfors, Eero Laakkonen

**Affiliations:** a University of Turku, Department of Teachers Education, Rauma, Finland; b University of Turku, Department of Teachers Education, Turku, Finland

**Keywords:** Learning in a safety education programme, Safety education in the Finnish comprehensive education curriculum, Collaboration: teachers and experts, Safety competence, Teaching-learning methods

## Abstract

The Nouhätä! safety education programme has been organised in secondary schools in Finland for over 25 years. However, to date, it has not been systematically evaluated. The purpose of this quantitative survey is to provide information about good practices, benefits and limitations of the NouHätä! Programme; this has been done by answering the research question what variables explain pupils' safety competence after participating in a NouHätä! safety education programme? The results show that the best learning outcomes in safety education are achieved when training is organised in collaboration with teachers and safety experts. Practical training also seems to have a significant impact on the safety competence of pupils. The results suggest that background variables like school success and the sources of safety knowledge affect the level of pupils' safety competence. The results of the study can be used to develop the programme and other safety education programmes for children and young people.

## 1 Introduction

Accidents are unnecessary disruptions to everyday life. They can cause many kinds of damage which can influence an individual's ability to function and work, and even cause the loss of human life. Accidents may weaken people's sense of safety and have national economic impact [1]. Accidents are the fourth most common cause of deaths in Finland. Finland has a total population of around 5.5 million people and, in 2020, 2373 people died due to accidents, including unintentional alcohol poisoning [2]. About 90 percent of unintentional deaths and about 80 percent of accidents leading to disability occur at home and during leisure time [3,4].

To reduce these numbers, national goals for preventing incidents and accidents have been drafted in different social strategies in Finland. The Target Programme for the Prevention of Home and Leisure Injuries 2021–2030 [5] produced by the Ministry of Social Affairs and Health, and The Action Plan for Incident Prevention for the Rescue Services produced by the Ministry of the Interior [6] support each other and guide the promotion of safety and safety education in a multidisciplinary cooperation. The programmes emphasise the importance of safety skills in accident prevention. The action plan of the Ministry of the Interior in particular highlights the development of safety competence for children and young people as one of the vital goals which influence accident prevention [6].

### 1.1 Safety competence education for injury prevention

Safety is an important value in Finnish comprehensive education (grades 1–9, ages 7–16). In the Finnish National Core Curriculum for basic education [7], goals related to safety education are emphasised in the curriculum content of many school subjects, such as, environmental education, technology education, physical education, and health education. Safety-related curriculum content appears in the context of 11 different subjects at different grade levels in total. Safety-related goals in the curriculum and in other normative documents mainly target learning to prepare for hazards, safely behaviour in everyday life, recovery from safety deviations and taking ownership of safety [8]. Moreover, the basic education curriculum also enables cooperation with communities and experts from outside the school [9].

Although the concept of competence is generally used to define professional competencies [10,11], it is also suitable for describing the totality of knowledge, skills, attitudes, and willingness related to safety. In safety education, the aim is to develop the safety competence of pupils [8,12]. Safety competence refers to the knowledge and skills needed to perform a task or solve a problem. It can also include attitudes and values that influence behaviour. It also refers to a person's ability to apply their knowledge and skills to different situations [13,14]. When an accident threatens, a person must be able to act in the unexpected situation and apply knowledge and skills in an appropriate way [8,15]. Practical skills training plays a very important role in the development of safety competence [16,17]. In safety research, the totality of these different factors has often been examined using the KAP model, this acronym refers to the words: knowledge, attitudes, and practices [18,19].

Finnish schools have strongly relied on cooperation with authorities and organisations in safety education [17,20]. Accident prevention is a statutory task of the rescue services [21]. Therefore, children and young people are one of the most important target groups for public education in fire safety. The accident prevention action plan of the rescue services requires that the rescue services be in contact with every child four times before the age of 25. Additionally, the action plan encourages the use of versatile methods in safety education work and cooperation with different parties [6].

In Finland, teachers are free to use experts from outside the school to assist them in their teaching. The normative guidance in basic education encourages adopting learning environments and working methods that promote interaction, participation, and joint knowledge building. This also enables collaboration with communities and experts outside the school, which adds authenticity and flavour to school work [9].

According to Somerkoski, Kärki and Lindfors [22] the use of experts in safety education motivates pupils, deepens their understanding of the topic, and provides functional activities. One challenge related to the use of experts, however, is the structural rigidity of the school day, which makes it difficult to book and arrange times with outside parties. Responsibility issues can also sometimes be unclear. Even when working together, it is important for teachers to recognise their own responsibility for teaching and for their pupils. Collaboration with outside experts and agencies can complement, though not replace, the efforts of the school.

### 1.2 NouHätä! safety education programme

An example of long-term cooperation among schools and rescue services is the Finnish NouHätä! Safety Education programme aimed at 8th graders (14–15 years old pupils). The programme, carried out in collaboration between the Rescue Service and the Finnish National Board of Education, has been organised regularly since 1996 and reaches around 45 000 pupils annually [23]. In 2020, a total of 62 115 pupils studied in the 8th grade in Finland [3]. Therefore, the programme was able to reach 72 % of the pupils of the entire age group.

**Fig. 1:**
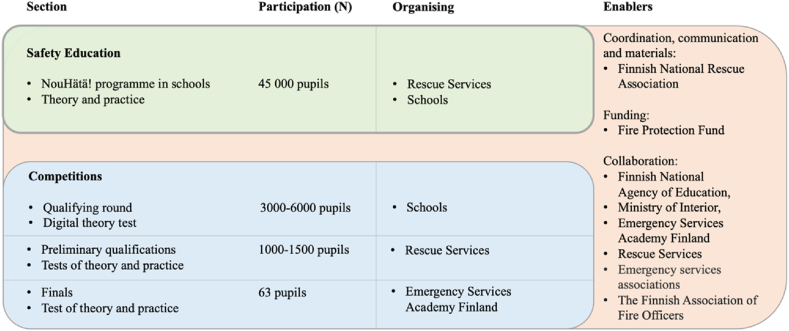
The overall structure of the NouHätä! programme (adapted from Mertasalmi & Kivelä [23]).

The goal of the NouHätä! programme is to teach how to prevent fires and other accidents, how to respond to emergencies and how to prepare for social disruptions. In the programme, teaching is usually carried out in interprofessional collaboration between the rescue service personnel and schoolteachers. The materials, such as videos and exercises, are provided by the Finnish National Rescue Association. In the NouHätä! programme, teaching consists of theory and various practical and online exercises. Practical training, such as first aid and fire extinguishing exercises, deepens the theoretical knowledge of the pupils. The NouHätä! programme also includes a voluntary competition. Success in the competition is determined by knowledge, skills and teamwork. The Finnish National Rescue Association coordinates the NouHätä! programme, and it is implemented with funding from the Fire Protection Fund [23]. The overall structure of the NouHätä! programme is described in Fig. 1.

Even though the Nouhätä! programme has been implemented for more than a quarter of a century, it has received little scholarly attention. Rekola, Itkonen & Saine-Kottonen [24] examined the effectiveness of safety education by the rescue services and the NouHätä! programme on the safety competence of young people. Pedak, Mankkinen and Kolttola [25] published an evaluation study on the effectiveness of fire safety campaigns, such as the NouHätä!-programme.

Somerkoski and colleagues [22] examined the experiences, challenges, and opportunities associated with safety interventions implemented by external partners. One of the interventions examined in the study was the NouHätä! programme. In addition, the NouHätä! programme has been the subject of several theses published at universities of applied sciences*,* investigating e.g. attitudes and safety awareness among young people and good educational practices. The annual monitoring carried out by the programme organisers is mainly based on statistical information about the development of the number of schools and pupils involved in the programme.

All the educational materials of the NouHätä! programme can be found in an open online materials bank. The teachers and trainers’ section contain teaching materials for lessons, various exercises, video lectures and teaching videos. The pupils' section contains a variety of online exercises to support the lesson materials [23]. In addition to being easy to update, the advantage of digital learning materials is that they can be accessed by anyone, anywhere, anytime. Pupils can also choose options they prefer from the self-study materials [26]. Another advantage of digital learning materials is that they are motivating; materials in digital formats are likely to be more appealing to pupils than materials on paper [27].

### 1.3 Aim and research problems of the study

When assessing the safety competence and learning outcomes of pupils after a programme, it is important to consider not only the use of different teaching methods and materials but also other factors that influence learning. PISA surveys in Finland have found gender gaps in reading, mathematics, and science [28]. Previous studies on students' safety competence have also found gender differences in attitudes and performance - for example, girls generally have a more positive attitude to safety [[29], [30], [31]]. Moreover, socio-economic factors, as well as pupils' school performance, have also been found to have an impact not only on their knowledge and skills but also on their perceived sense of safety. In the WHO School Student Survey, 80 % of students who did very well in school and 60 % of those who did less well felt safe [32]. Moreover, as in other areas of knowledge, the world outside the school is an important learning environment for safety skills. In the studies examining Finnish safety education, young people recognised learning about safety issues not only at school but also at home, in the media, and through hobbies [24,33].

There are relatively few studies in the field of education that examine the causality between the learning outcome and the time spent teaching. The impact of time used on learning outcomes is not linear, but after a certain level, the benefits of increasing time decrease. Students of different abilities and socio-economic backgrounds respond differently to extended or intensified instruction [34]. Välijärvi [35] has stated, that in the PISA studies the link between time spent learning and the outcomes of learning are not unequivocal. There are significant differences between school systems in the amount of time pupils spend studying different subjects in and out of school. Relative to learning outcomes, the efficiency of time used varies widely.

The aim of this study is to carry out a pilot survey exploring the safety competence of eighth graders (14–15-year-old pupils) after participating in a national safety education programme. The purpose of the study is to provide results that can be used in developing research-based safety education in secondary education. Our research interest is in the types of programmes where safety education is executed in collaboration with safety experts and schoolteachers. To this end, the study poses the following research questions:

• What is the safety competence level of pupils after participating in the NouHätä! programme?
• How was the NouHätä!-programme implemented in schools and how have the pupils learned safety information and skills from different sources?
• What are the variables that explain the safety competence of pupils after participating in the NouHätä! programme?

## 2 Materials and methods of the study

The target group of this study was 8th grade pupils who participated in the NouHätä! programme during the spring term of 2020. All survey data were collected through a single online Webropol survey. Before the actual survey was conducted, a group of 14 9th-grade students, 8 girls and 6 boys, participated in the pre-test. All these respondents had participated in the NouHätä!-programme a year earlier. The purpose of the pretest was to determine the comprehensibility and suitability of the survey claims for the subjects [36]. At the same time, the amount of time spent on the survey was estimated. Based on the pre-testing, questions in the question form were clarified and typographical errors were corrected. In the pre-test, it was also noticed that some of the students did the pre-test on their phone. After pre-testing, the actual test was optimized to work on phones.

The survey was sent to all principals of the schools involved in the NouHätä! programme. Principals were asked to distribute the survey to pupils who had participated in the programme. Information on the survey was also sent to the pupils' homes via the schools. Parental consent was sought to ensure that the pupils as minors had permission to take part in the study survey. Participation in the survey was voluntary.

Just as the survey was being prepared in March 2020, the restrictions due to the COVID-19 pandemic were imposed, and pupils in Finland as elsewhere transitioned to distance learning. The assumption was that pupils would answer the survey independently during the distance learning period. Therefore, the electronic form was optimized for mobile use and the instructions were made as unambiguous as possible. Additionally, the instructions were published in video format to improve data collection. The survey was launched in early May, but due to distance learning, the response rate was initially low. When schools returned to face-to-face teaching in May, the survey response period was extended, and reminders were sent to schools. Subsequently, the response rate increased.

The response variable in the study was the total score on the safety test. The test assessed pupils' safety competence with 12 statements based on the educational content and objectives of the NouHätä! programme [23]. The items were designed to measure knowledge, skills, and attitudes according to fire safety, first aid skills and common safety issues. The survey requested everyone to respond independently according to their current level of knowledge. The response options for all statements were ‘Yes’, ‘I don't know’ and ‘No’. The answers were scored so that each correct answer earned one point. An incorrect answer and the ‘I don't know’ option were worth zero points. After scoring, the total test score for each pupil was calculated. The Kuder-Richardson 20 coefficient was calculated for the safety test, yielding a value of 0.78. This result indicates a reasonably high level of reliability in the test scores. A coefficient closer to 1 suggests that the individual items or questions in the test are more consistent [36,37].

In addition to testing safety competence, the variables included in the survey were the use of methods and materials, use of time, implementation of the safety education in the NouHätä! programme, respondent's gender, school performance and sources of safety knowledge and skills.

The use of materials and methods for training and self-study in the NouHätä! programme was explored using a survey section that considered all the methods and materials available in the programme, such as, educational videos or practical skills training. These answer options were also ‘Yes’, ‘No’, and ‘I do not know’.

The implementation of the NouHätä! training at schools was examined with three different statements: 1) training was implemented by both the rescue service and the school, 2) by one or more rescue service representatives, and 3) by one or more teachers from the school. Respondents could only choose one of these options.

The use of time in the Nouhätä! programme was examined from three different perspectives. First, respondents were asked to estimate how many hours they thought they had spent on safety education during the NouHätä! programme. Next, the respondents were asked how many hours they had spent learning about safety on their own during the programme and finally, how many hours they would have liked to spend in school on the NouHätä! programme. The response options were pre-sorted into four categories.

The previous school performance of the respondents was examined by asking about the average of their previous school report with an accuracy of one decimal place. The responses followed a normal distribution (low skewness and kurtosis, −0.51 and 0.24 respectively), and the mean score was 8.17. The rating scale in Finland is 4–10.

Respondents were also asked to identify different sources of safety knowledge and skills. There were seven different options to choose from; school, home/relatives, hobbies, friends, media, independent study, games and other. The response scale was a five-point Likert scale where 1 meant “not at all” and 5 meant “very much".

A total of 1410 comprehensive school 8th grade pupils responded to the survey. Twelve responses were deleted from the data as either all the options had been left blank, or all the questions had been systematically answered in the same way. The final number of participants was 1398 (N = 1398). Of all the respondents, 701 reported being girls and 639 boys. Fifty-one respondents chose the option “Other/I do not want to answer,” and 7 did not answer the gender question.

All questions and answer options of the survey can be found in Appendix 1.

The survey results were first examined using a descriptive statistical methods. Correlation analyses were used to examine the relationship between the explanatory variables and the safety test results. A statistical regression model was also created from the data to examine the linear dependence between the explanatory variables and the variable to be explained [38]. In the descriptive analyses, the original forms of the variables were used. In the correlation and regression analyses, the categorical variables were recoded so that they were more suitable for analyses. Analyses were performed using IBM SPSS STATISTICS 28.

## 3 Results

### 3.1 Pupils' safety competence after participating in the NouHätä! programme

The response variable in the study was the total score of the safety competence test on fire safety, emergency skills, and common safety issues. The test assessed pupils' safety competence with 12 statements: knowledge (variables 1–4), skills (variables 5–8) and attitudes (variables 9–12). All 1398 pupils answered this test. Table 1 shows the percentage distribution of responses.

**Table 1:** The results of the safety test variables in national safety programme NouHätä for 8th graders (N = 1398).

Variable	Correct answers	Wrong answers	I do not know
1. The most dangerous thing about a house fire is heat.^a^	78.8	9.2	11.8
2. The rescue services are responsible for safety immediately if a fire occurs at school.^a^	34.9	38.0	26.3
3. About 500 000 accidents occur in Finland every year.^a^	10.6	43.9	44.8
4. On average, more people have died by drowning than in fires in Finland during recent years.	29.2	20.3	49.5
5. I could put out a burning rubbish bin with a powder extinguisher.	70.1	6.8	22.5
6. Cardiac arrest is detected by palpating the pulse from the carotid artery.^a^	12.8	67.4	19.3
7. A grease fire can be safely extinguished with water.^a^	84.9	2.9	11.6
8. The most important thing in CPR is effective artificial respiration.^a^	34.1	30.6	34.3
9. I would always help a person in an emergency, even if I did not know him.	73.3	4.0	21.9
10. I do not think I need safety knowledge and skills in the future.^a^	65.9	7.3	26.0
11. I think it is important to have a working smoke alarm at home.	84.0	3.0	12.0
12. Photographing an accident site may be detrimental to helpers or victims.	65.1	10.5	23.2

a Correct answer is NO. In other variables the correct answer is YES. KR20 with 12 variables .78.

For each correct answer, the respondent received one point. An incorrect answer and the “I don't know” option received zero points. Although the attitude questions do not have the same absolute right or wrong answers, they were also scored so that a response that could be classified as a positive safety attitude received a point. Only two respondents scored a total of 12 points on the test. A score of 0 was obtained by 80 respondents. The emphasis on the weaker end of the scale is due to the fact that the test was not easy, and some respondents had answered “I don't know” to many statements. The mean test score was 6.40 with a standard deviation of 2.61.

In the results, attention was paid to the statement on extinguishing a grease fire. Up to 84.9 % of the respondents knew the correct answer and only 2.9 % gave the wrong answer. There was also a good response rate to the item claiming that heat is the most dangerous factor in a fire. Altogether 78.8 % of respondents answered correctly. Questions on initial firefighting skills and readiness to help also scored highly. On the other hand, the results show that the level of competence in first aid was low. To describe the competence in detail, the total score was reclassified into six categories: poor (from 0 up to 4.8), passable (from 4.8 to 6), fair (from 6 to 7.2), satisfactory (from 7.2 to 8.4), good (from 8.4 to 9.6) and excellent (from 9.6 to 10).

Table 2 shows the distribution of results across these categories. Three fifths of the pupils did not score more than fairly in the test.

**Table 2:** Distribution of scores in the safety competence test.

	Frequency	Percent	Cumulative Percent
Poor	260	18.6	18.6
Passable	321	23.0	41.6
Fair	270	19.3	60.9
Satisfactory	270	19.3	80.2
Good	190	13.6	93.8
Excellent	87	6.2	100.0
Total	1398	100.0	

### 3.2 Materials and methods used in the NouHätä! programme

The Finnish National Rescue Association created the web-based learning material for the NouHätä! programme. The material consisted of videos, leaflets and lessons. Teachers involved in the NouHätä! programme could freely choose materials to implement from these for the NouHätä! lessons at their schools. Pupils also used these materials for self-directed learning outside school lessons.

The pupils' responses (N = 1373) showed the most used methods in the NouHätä! programme were the educational videos (62.4 %) and the practical skills training (61.3 %). The videos where YouTube stars were used to promote the campaign came last (27.5 %). The response which received the most ‘I don't know’ responses (49.1 %) was the use of lesson plans by the teachers. The percentage distribution of responses is shown below in Table 3. The column on the right shows the percentage of individual blank responses (the respondent had answered one of the other options in the question battery but not this one).

**Table 3:** Use of materials and methods in NouHätä! programme according to the views of the 8th graders (N = 1373).

Use of materials and methods in the NouHätä! programme	Yes	No	IDK	Missing
Educational videos (e.g. fire extinguishing)	62.4	10.1	26.2	1.2
Practical skills training (CPR, fire extinguishing, emergency evacuation etc.)	61.3	12.2	25.6	0.9
Video lectures (mini-lessons or full lesson)	45.7	22.0	31.2	1.1
Powerpoint-slideshow	45.0	17.4	35.9	1.7
Online exercises and games	37.8	28.7	32.9	0.6
Lesson plans for teachers (safety-related lessons for use in school)	28.3	21.1	49.1	1.5
Videos from YouTube stars (e.g. Biisonimafia, Soikku, Tume etc.)	27.5	42.2	29.4	0.9

Note: IDK = I don't know.

### 3.3 Implementation of the NouHätä! safety education programme

Three different statements were used to investigate how the NouHätä! training was implemented in schools. The total number of responses to this question was 1290. Over half of the pupils indicated that the teaching was provided by both the rescue service and the school, 26 % by one or more rescue service representatives, and 22.6 % by one or more teachers from their own school.

The use of time in the NouHätä! programme was examined from three perspectives. First, the respondents were asked to estimate how many hours they thought they had spent on safety education during the NouHätä! programme. Next, the respondents were asked how many hours they estimated to have spent learning about safety on their own during the programme and finally, how many hours they would have liked to have spent in school on the NouHätä! programme. The response options were pre-sorted into four categories. There were 1338 responses to this question. The percentage distribution of responses is shown below in Table 4. The column on the right shows the percentage of individual blank responses. The results show that the most common response related to time spent being taught safety education content was 2–4 h (38.3 %), but almost as many (34.7 %) spent less than 2 h being taught. Most respondents (69 %) reported spending less than 2 h on self-study.

**Table 4:** The use of time in NouHätä! programme (N 1338).

The use of time in the NouHätä! programme	under 2 h	2–4 h	4–8 h	over 8 h	Missing
How many hours have you spent participating in safety education provided by a school teacher or rescue personnel during the NouHätä! programme?	34.7	38.3	20.9	5.2	1.0
How many hours have you spent learning about safety on your own during the NouHätä! programme?	69.0	19.5	7.5	2.2	1.9
How many hours would you have liked to spend in school on the NouHätä! programme?	32.1	39.2	20.0	6.7	2.0

### 3.4 Sources for acquiring safety knowledge and skills

While the focus of survey was on the NouHätä! programme, pupils (N 1393) were also asked to identify different sources for acquiring safety knowledge and skills. The school (mean 3.7), home and relatives (3.7), and the media (mean 3.4) were seen as the most important sources for acquiring safety knowledge and skills. The response option ‘Home or relatives’ received the highest number of “Very much” responses and the lowest number of “Not at all” choices.

Games received the lowest scores (mean 2.6), but almost the same average score was also achieved by friends as a source of safety knowledge and skills (2.6).

In the last section, pupils were given the opportunity to select another option as a source of safety knowledge and to specify their answers in the open message box. Many of the answers written in this field, such as “movies, YouTube and Voluntary fire brigade activities” could be linked to the options presented earlier, but “moped driving school, camps, holiday trips, kindergarten, job training, and children's club leader training” emerged as new sources of safety knowledge and skills.

The percentage distribution of responses is shown below in Table 5. The column on the right shows the percentage of individual blank responses.

**Table 5:** Sources of safety knowledge or skills (N 1393).

Sources of safety knowledge or skills	1 Not at all	2 Very little	3 Slightly	4 Quite much	5 Very much	*Mean (1–5)*	missing
Home or relatives	1.7	5.6	25.0	53.3	13.6	*3.7*	0.9
School	2.1	2.5	23.7	63.5	7.5	*3.7*	0.9
Media (TV, radio, magazines, social media etc.)	3.4	9.5	32.7	44.0	9.5	*3.4*	0.8
Independent study	8.5	16.1	38.4	30.4	5.5	*3.0*	1.1
Hobbies	17.3	18.4	33.4	20.9	8.8	*2.8*	1.2
Friends	13.9	26.1	42.4	13.4	2.8	*2.6*	1.4
Games (videogames, board games etc.)	19.7	23.6	33.3	17.7	5.2	*2.6*	1.4
Somewhere else (write in the box)	55.3	6.5	10.1	6.0	2.7	*1.4*	19.5

### 3.5 Modelling the relationships between variables

The survey results described above provide answers to the specific questions and statements. We now know how pupils have responded to various questions on safety and teaching methods. We also know how the results of the safety test are distributed among the respondents. However, to answer the last research question, we still need to look at the associations between the safety competence test scores and the explanatory variables.

Multivariate linear regression analysis with the stepwise procedure was conducted with the total test scores as the dependent variable and 21 other explanatory variables. In the stepwise procedure, independent variables are included in the model one by one, until only the set of statistically significant explanatory variables remain in the final model. As a result, the final model included 14 predictors.

Prior to conducting the linear regression analysis, certain variables were modified to enhance their interpretability. In the gender variable, the frequency of the category 'other/I don't want to answer' was low, so it was omitted from the analysis and the gender-variable was applied as dichotomous (female = 1 and male = 0). Additionally, within the materials and methods section, responses were recoded into a dichotomous format (Yes = 1 and No/I don't know = 0). The education implementation variable had three categories, so for the regression analysis it was dummy-coded with the response category “schoolteacher” as the reference category. Descriptive statistics and correlations between variables included in the analysis can be found in Appendix 2. Pearson, point-biserial and phi correlations were used according to the measurement scales of the variables.

Table 6 below shows the variables that collectively explain the pupils' safety competence. The R^2^ value of 0.342 shows that 14 predictor variables explain 34 % of the variance in success on the safety test. The table reports the regression coefficients B with standard errors, standardized coefficients beta, test-statistics t, p-values for statistical significance and 95 % confidence interval for coefficients B.

**Table 6:** Explanatory variables of a linear regression model explaining the safety skills of 8th graders (N = 1216).

	B	SE	Beta	t	p	95 % CI
(Constant)	0.56	0.63		−0.90	0.370	[−1.80, 0.67]
School performance	0.60	0.07	0.22	8.54	<0.001	[0.46, 0.74]
Materials and methods: Practical skills training	0.90	0.15	0.17	6.18	<0.001	[0.61, 1.18]
Materials and methods: Educational videos	0.68	0.16	0.13	4.30	<0.001	[0.37, 0.98]
Materials and methods: Online exercises and games	0.45	0.13	0.09	3.36	0.001	[0.19, 0.71]
Materials and methods: PowerPoint slideshow	0.34	0.13	0,07	2.60	0.001	[0.08, 0.60]
Materials and methods: Videos from YouTube stars	0.33	0.14	0.06	2.31	0.021	[0.05, 0.60]
Materials and methods: Video lectures	0.29	0.13	0.06	2.14	0.032	[0.02, 0.55]
Use of time: How many hours have participated in training	0.29	0.07	0.11	3.89	<0.001	[0.14, 0.44]
Use of time: How many hours of independent study	−0.33	0.09	−0.10	−3.63	<0.001	[−0.51, −0.15]
Sources of safety knowledge or skills: independent study	0.23	0.06	0.10	3.60	<0.001	[0.10, 0.36]
Sources of safety knowledge or skills: Home or relatives	0.20	0.08	0.07	2.59	0.001	[0.05, 0.35]
Sources of safety knowledge or skills: Friends	−0.34	0.07	−0.13	−5.16	<0.001	[−0.47, −0.21]
Sources of safety knowledge or skills: Somewhere else	−0.15	0.05	−0.07	−2.95	0.003	[−0.25, −0.05]
Implementation of the NouHätä! Safety education programme as provided by both the rescue service and the school (collaboration vs reference category school only)	0.33	0.13	0.07	2.54	0.011	[0.07, 0.58]

R^2^ = 0.342.

The leading sign of the coefficients indicates which end of the response scale the result is weighted towards. Thus;

1) A positive regression coefficient for the school performance (B = 0.60) suggests that a good certificate predicted better test performance.
2) The positive B-coefficients of the six variables concerning materials and methods indicate that use of the practical training and educational videos in particular as well as online exercises, PowerPoints, and videos of YouTube stars had a positive effect on test performance.
3) In relation to the use of time, the regression model ended up with two variables, one with a positive and one with a negative coefficient. The result can be interpreted as meaning that the more the pupils could participate in the NouHätä! lessons at school (B = 0.29), the better they did on the test. On the other hand, it seems that those pupils who spent more time on self-study (B = −0.33) did less well.
4) For the variables on learning safety knowledge and skills, the results show that independent study (B = 0.23) and acquiring safety knowledge and skills from home or relatives (B = 0.20) were positively related to test scores. Instead, learning safety knowledge and skills from friends (B = −0.34) or elsewhere (B = −0.15) was inversely related to test performance. This means that respondents who reported acquiring little or nothing about safety from these latter sources did better on the test.
5) In relation to how the responsibilities related to the programme implementation were divided, a positive coefficient (B = 0.33) refers to the benefit of the collaboration of a rescue expert and a schoolteacher (compared to the reference category ‘Only the schoolteacher’).

## 4 Conclusions

### 4.1 Discussion and limitations

During the 25-year history of implementing the NouHätä! programme in Finland, the present study represents the first peer-reviewed research to focus exclusively on the programme nationwide. The aim was originally to conduct an overall study of the programme, and the survey was sent to all schools participating in the programme. However, the distance learning period during COVID-19 and having only a short period of face-to-face teaching before the summer break made the data collection challenging. From the perspective of all participants in the programme, the sample represents only 3.5 percent. In contrast, the survey data was sufficiently wide (N 1398) and the responses were reasonably normally distributed, allowing for a robust statistical description and analysis. The results of the safety test can also be considered fairly reliable (KR20 0.78). The explanatory power of the regression model variables was 34 %, which can be considered strong compared to social and behavioural sciences in general [36,39]. However, all the factors influencing the variable under study were impossible to know or include in the analysis, and as researchers in pedagogy, we are fully aware of the challenges of demonstrating causality [40]. Furthermore, the learning outcomes were only measured once after the pupils participated in the programme which does not provide information on how permanent the learning outcomes will be. In addition, a control group was not used. However, when examining a large numbers of pupils, as in this study, a survey can effectively be used to investigate pupils' knowledge and attitudes [36]. However, a survey is not the most reliable method for assessing practical skills. Instead, the skills should be assessed by observation, which was not possible due to the pandemic and the large number of pupils. Even though the pandemic posed challenges to the implementation of this study, it is noteworthy that the programme itself was organised before the pandemic restrictions were imposed, which allowed for a reliable and comparable study design.

It must be noted, that the results are the pupils' own reflections on their own performance, attitudes, knowledge and skills. The study succeeded in its aim of examining and explaining correlations among explanatory variables related to the safety competence of 8th graders in secondary education after participating in the NouHätä! programme. In order to achieve this, a comprehensive safety competence test (Table 1) based on the NouHätä! programme goals was utilised. Additionally, the study succeeded in investigating the implementation of the NouHätä! programme and exploring pupils’ views on their sources of safety knowledge and skills. Despite the limitations the of the study the results provide a research-based path for developing the NouHätä-programme, which might also be used in developing other safety programmes as well. In the future, more support for the results could be obtained by repeating the study or by targeting the sample more accurately; for instance this could be done on geographical grounds using discretionary sampling. Moreover, a similar survey could be conducted with a control group as a pre- and post-test. In the future, practical skills could also be assessed using a qualitative approach with observations.

### 4.2 Summarising the results and their implementation

In this survey study, the safety competence of lower secondary education pupils in comprehensive education was studied as a combination of attitudes, knowledge, and skills particularly as they relate to fire safety, first aid and common safety statements; their competence was evaluated by the pupils themselves. The study reveals that after the NouHätä! programme pupils′ fire safety competence was generally at a good level (Table 1). The results regarding fire safety were somewhat better than the results regarding first aid. From the perspective of attitudes, most pupils had a positive attitude toward safety. However, the results of the safety competence test overall were diverse (Table 2) and raised concerns. Only two pupils of 1398 tested received full points and only one fifth achieved a good or an excellent level (Table 1). Many pupils answered ‘do not know’ to the statements and one fifth of the pupils scored poorly. The regression analysis revealed that a key variable explaining safety competence is the pupils' overall performance in school. A pupil who performs well in school also performed well in the safety competence test (Table 6). The active use of programme materials and methods, such as the practical skills training and the use of educational videos, were also strongly associated with better scores in the safety test.

The results firmly revealed that the educational videos and the practical skills training in developing safety competence were those most used according to the pupils (Table 3). A rather surprising find was that the videos produced by YouTube stars were not used or considered as part of the teaching materials. Almost half of the pupils said that YouTube videos had not been used and almost a third did not know if they had been used. The materials and methods were widely represented in the regression model (Table 6). In addition to the practical skills and educational videos, the online exercises and games were mostly responsible for the safety competence learning of pupils and were associated with better scores on the safety test.

When considering the way the education was implemented, the most common way to organise NouHätä! training was to organise it through cross-institutional collaboration between the school and rescue services. In practice, the teaching could be carried out in three different ways; by the school, by the rescue services or as a collaboration of both [23]. Collaboration produced proportionally significantly better test results and fewer poor results than when the programme was carried out by the school or the rescue service alone (Table 6). There seem to be an unspecified quality factor related to collaboration that leads to better learning outcomes. However, this data does not allow for a deeper analysis of the collaboration, but it can be argued that the collaboration adds variety and interest to the teaching. It might be that where a representative of the rescue service was involved, the teaching was more likely to include practical training [22]. On the other hand, the fact that safety education involves not only the schoolteacher but also an external expert suggests more time is likely to be spent on the training. In future studies, a multiple case study or comparative research design might provide a deeper understanding. However, this kind of collaboration in safety education is an issue that should be considered while developing the Nouhätä or other safety programmes.

Pupils viewed the school, home and relatives, and the media as the most important sources for learning safety knowledge and skills (Table 5). In addition, pupils highlighted some other sources of learning, such as driving school, camps and job training. In contrast, learning safety knowledge and skills from friends or elsewhere and the high level of self-learning in the NouHätä! programme had a negative impact on the test result. A gender difference was also found with girls scoring better than boys on average. In this respect, the results were in no way surprising, as the findings related to the link between school performance and gender [28] and safety skills have been highlighted in previous studies as well [29,30]. However, in the regression analysis, gender as a variable did not add to the explanatory power (Table 6).

Considering that safety competence consists of knowledge, skills, attitudes and willingness to act [8,15], our result reinforce the evidence for proposing the use of practical methods in safety education [16,17]. Practical skills training seems to play a key role in learning safety-related content (Table 3, Appendix 2, Table 6). This is important to bear in mind when planning and executing safety education. Experiential learning improves sustainability of learning outcomes [22] and, ultimately, it is the practical skills that are essential in safety competence. Awareness of the number of people who die in fires each year may influence attitudes to fire safety, but basic extinguishing skills, responding to emergencies and rapid evacuation are crucial to surviving in an accident. It is also useful to teach accident prevention in a practical way [15,20]. Simultaneously, the study also observed the connection between school performance and safety competence. Those who performed well at school also did well in the safety competence test and vice versa. This leads us to question whether safety education takes into account the needs of different learners, for instance, in terms of varied learning materials. Safety is a necessity for everyone and everyone needs safety competence in their life, which is why it is important that everyone has an equal opportunity to learn it, regardless of any learning difficulties or other challenges that may impact the learning process. This topic, as well as matters related to collaboration and co-teaching should be further explored in the future.

## Ethical statement

The guidelines of the ethical principles of research with human participants and ethical review in the human sciences in Finland [41] were carefully evaluated. No review and/or approval by the Ethics Committee was required for this study.

## Data availability statement

Data will be made available on request.

## CRediT authorship contribution statement

**Mikko Puolitaival:** Writing – original draft, Project administration, Methodology, Investigation, Conceptualization. **Brita Somerkoski:** Writing – review & editing, Writing – original draft, Supervision, Conceptualization. **Eila Lindfors:** Writing – review & editing, Validation, Supervision, Conceptualization. **Eero Laakkonen:** Writing – original draft, Methodology, Investigation, Data curation.

## Declaration of competing interest

The corresponding author of this article, Mikko Puolitaival, works at the Rescue Department alongside his PhD studies. It is worth noting that rescue services are one of the partners involved in the NouHätä! programme; however, Mikko himself is not directly engaged in the teaching of the program. Mikko has also received a grant from the Fire Protection Fund (SMDno-2017-2035). The funder has had no role in the subject or implementation of the study. We wish to disclose that we have no other conflicts of interest to declare.

## Footnotes

Appendix A Supplementary data to this article can be found online at https://doi.org/10.1016/j.heliyon.2024.e28099.
