# Supplementary material for: Safety competence promotion in secondary education – A case of the Finnish NouHätä! Programme
Source: Heliyon. 2024 Apr 12;10(8):e28099. doi: 10.1016/j.heliyon.2024.e28099 (PMC11043864; doi:10.1016/j.heliyon.2024.e28099)
Supplement: Multimedia component 1 [file mmc1.docx]

The average of your last certificate as accurately as you remember (Use the slider to select a value)

The rescue services are responsible for safety immediately if a fire occurs at school.

Appendix 1. NouHätä! questionnaire

1. Gender

Female

Male

Other / I do not want to answer

2.

4

4 10


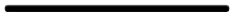


3. How much have you learned about safety knowledge and skills through:


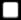


Erittäin

Not at all vähän Slightly

School?

Home/relatives?

Hobbies?

Friends?

Media (tv, radio, magazines, social media
etc.)?

Independent study?

Games (videogames, boardgames etc.)?

Somewhere else (write in the box)?

4. Answer each statement according to your knowledge and skills.

1) The most dangerous thing about a house fire is heat.

2)

3) About 500,000 accidents occur in Finland every year.

4) On average, more people have died in drowning than in fires in Finland in recent
years.

5) I could put out a burning rubbish bin with a powder extinguisher.

6) Cardiac arrest is detected by palpating the pulse from the carotid artery.

Quite much Very much

Yes I do not know No

, even if I didn't know him.

12) Photographing an accident site may be detrimental to helpers or victims.

Have the following materials and methods been used in your NouHätä!-teaching or have you used them in

NouHätä! was organised by one or more teachers from your own school

7) I would always help a person in an emergency

8) A grease fire can be safely extinguished with water.

9) I don't think I need safety knowledge and skills in the future.

10) I think it is important to have a working smoke alarm at home.

11) The most important thing in CPR is effective artificial respiration.

5. Use of methods and material in NouHätä!-programme

self-study.

Kyllä

Powerpoint-slideshow

Online exercises and games

Video lectures (mini-lessons or full lesson)

Educational videos (e.g. fire extinguishing)

Videos from Youtube stars (e.g.
Biisonimafia, Soikku, Tume etc.)

Practical skills training (CPR, fire
extinguishing, exit from the building etc.)

Lesson plans for teachers (safety related
lessons for use in school)

6. Use of time in the NouHätä!-programme

under 2 hours

How many hours you have you participated
in safety education provided by a school
teacher or rescue personnel during the
NouHätä!-programme?

How many hours have you spent learning
about safety on your own during the
NouHätä!-programme?

How many hours you would liked to spend in
school on the NouHätä!-programme?

7. Organising of the NouHätä!-training:

Yes I do not know No

I do not know No

2-4 hours 4-8 hours over 8 hours

NouHätä! was organised by one or more rescue service representatives

NouHätä! was organised by both the rescue service and the school
